# Spatial and Seasonal Variations of Sedimentary Organic Matter in a Subtropical Bay: Implication for Human Interventions

**DOI:** 10.3390/ijerph17041362

**Published:** 2020-02-20

**Authors:** Xuan Lu, Fengxia Zhou, Fajin Chen, Qibin Lao, Qingmei Zhu, Yafei Meng, Chunqing Chen

**Affiliations:** 1 College of Ocean and Meteorology, Guangdong Ocean University, Zhanjiang 524088, China; luxuan077@163.com (X.L.); fxzhou@yic.ac.cn (F.Z.); zhuqingmei1987@163.com (Q.Z.); mengyafei199001@sina.com (Y.M.); chenchunqing1221@163.com (C.C.); 2 Guangdong Province Key Laboratory for Coastal Ocean Variation and Disaster Prediction, Guangdong Ocean University, Zhanjiang 524088, China; 3 Marine Environmental Monitoring Center of Beihai, State Oceanic Administration, Beihai 536000, China; laoqibin@163.com

**Keywords:** sedimentary organic matter, stable isotopes, seasonal variations, anthropogenic influence, Zhanjiang Bay

## Abstract

Elemental (total organic carbon (TOC) and total nitrogen (TN)) and stable carbon and nitrogen isotope compositions (δ^13^C and δ^15^N, respectively) in the surface sediment of Zhanjiang Bay (ZJB) in spring and summer were measured to study the spatial and seasonal changes of organic matter (OM) and assess the human-induced and environment-induced changes in the area. The OM in the surface sediment of ZJB was a mixture of terrestrial and marine sources, and was dominated by marine OM (54.9% ± 15.2%). Compared to the central ZJB, the channel and coastal ZJB areas had higher δ^13^C and δ^15^N values, higher TOC and TN concentrations, and lower TOC/TN ratios, indicating higher primary productivity and higher percentages of marine OM in the latter two subregions. Mariculture activities, sewage inputs, and dredging were responsible for these phenomena. Clear seasonal variations in OM were observed in ZJB. The average proportions of terrestrial OM in summer increased by 10.2% in the ZJB channel and 26.0% in the coastal ZJB area compared with those in spring. Heavy rainfall brought a large amount of terrestrial OM into the channel and coastal ZJB areas, leading to the increase of the terrestrial OM fraction in these two subregions in summer. In summary, anthropogenic influences had a significant influence on the spatial and seasonal variations of sedimentary OM in ZJB.

## 1. Introduction

Organic matter (OM) in sediment is a mixture of components from different sources. The concentration of total organic carbon (TOC) is used as a primary proxy to describe the abundance of OM [1]. Under the influence of both terrestrial and marine ecosystems, coastal ecosystems are among the most productive ecosystems on Earth [2,3]. More than 90% of global marine organic carbon is trapped in coastal sediments through complex physical, chemical, and biological processes [4,5]. The OM in the surface sediment of coastal areas is either derived from terrestrial inputs or is produced by marine phytoplankton. This matter can fuel food webs in these areas [3,6,7]. Understanding the distribution of OM in the surface sediments of coastal areas is vitally important in order to understand the carbon cycle there [8].

OM samples from various sources each have their own stable carbon and nitrogen isotopic composition (δ^13^C and δ^15^N, respectively) and total organic carbon to total nitrogen (TOC/TN) ratio (see [9] and references therein). For example, terrestrial OM, when not influenced by C_4_ plants, has δ^13^C values between −30‰ and −24‰, while the δ^13^C values of marine OM are higher, often between −22‰ and −18‰ (see [10] and references therein). These characteristics have been widely used to trace OM sources [10,11,12,13,14,15]. In addition, these OM parameters can be used to identify anthropogenic influences. For example, δ^15^N values near sewage outfalls are relatively high and can be used to trace sewage inputs [16]. Because of the complexity of the composition and source of OM, and the biogeochemical evolution that affects OM in coastal environments, most recent studies have employed a combination of δ^13^C and δ^15^N, along with the TOC/TN ratio, to trace OM sources [15,17,18,19]. Simple examination of only one or two of these parameters is insufficient because each of them may be subject to limitations [15,18].

The accumulation and preservation of OM in surface sediment is affected by multiple environmental factors, such as the redox conditions of the bottom water and sediment, primary productivity in the water column, the hydrodynamic environment for particle deposition, and the water depth [14,15,20,21,22,23,24]. In shallow coastal areas, the sedimentation rate of particulate organic carbon can be very high [22]. A weak hydrodynamic environment benefits fine particle deposition [15]. Low oxygen levels may prevent redox reactions of sedimentary organic matter (SOM) to some extent [25]. High primary production can lead to high SOM contents [14]. In shallow waters, OM undergoes little degradation while sinking, however in deep waters, a considerable fraction of the OM produced by primary producers is degraded as it sinks through the water column [26,27]. Primary production and oxygen levels in coastal areas often show seasonal variations, which may affect the accumulation of OM in sediment [28]. Fresh OM in surface sediment may not be preserved for a long time; much of it may decompose [29,30]. The deposition of OM from the water column may increase SOM, which may persist for several months [23]. Therefore, seasonal variations in environmental parameters may cause seasonal variations in OM parameters in various surface sediments. For example, OM parameters in the surface sediment of the San Francisco Bay (USA) channel have shown seasonal variations that follow the patterns of water column production, with chlorophyll peaks in the water column followed by later peaks in the sediment TOC and TN [23].

Coastal areas, including estuaries and bays, are important areas, not only for food production, but also for cultural and recreational purposes and for aesthetic value. However, these areas are heavily impacted by anthropogenic activities, such as aquaculture, dredging, and sewage disposal [27,31,32,33,34]. These activities have the potential to alter the distribution of SOM in coastal areas [10,34,35,36]. For example, increases in aquatic productivity as a response to sewage input contribute to an increase in sedimentary TOC concentration [27,37], and maricultural activities can increase particulate OM deposition and weaken hydrodynamic conditions, having a significant influence on the source, distribution, and preservation of SOM [15,38,39,40]. The natural balance between the production and decomposition of SOM in many coastal areas has been disturbed by anthropogenic activities [9]. Understanding the impacts of anthropogenic activities on the SOM is of great importance.

Many studies have examined the influence of seasonal variations in the environment and anthropogenic activities on SOM, and much useful information has been obtained. Lesen [23] studied the seasonal variations of SOM in San Francisco Bay, and found that the sediment TOC and TN were highest in spring and lowest in winter, which followed the pattern of primary production in the water column. Yang et al. [14] studied the SOM in surface sediments of the coastal northern Shandong Peninsula, China, and found that the summer TOC and TN concentrations in surface sediments were significantly higher than those in autumn and spring. High primary production and hypoxia conditions in summer, contrasting with sufficient dissolved oxygen in bottom waters in autumn and spring, were responsible for these seasonal variations in TOC and TN. Gao et al. [9] studied the geochemistry of organic carbon and nitrogen in surface sediments of coastal Bohai Bay, China, based on stable isotopic signatures and TOC/TN ratios, and found that the inputs of OM from anthropogenic activities had a more significant influence on parameter distributions than natural processes did. Pan et al. [15] studied the impacts of mariculture activities on sedimentary organic carbon (SOC) in Ailian Bay, China, by coupling δ^13^C and δ^15^N with TOC/TN ratio analyses. This study found that mariculture activities were a significant source of SOC, and also significantly influenced the distribution and preservation of SOC. However, most prior studies investigating the influence of anthropogenic activities and seasonal environmental variations on SOM have been undertaken in temperate zones [9,14,15,23], and much less is known about subtropical bays. Besides, to our knowledge, the influence of seasonal variations in rainfall on SOM has rarely been documented in previous studies.

**Figure 1:**
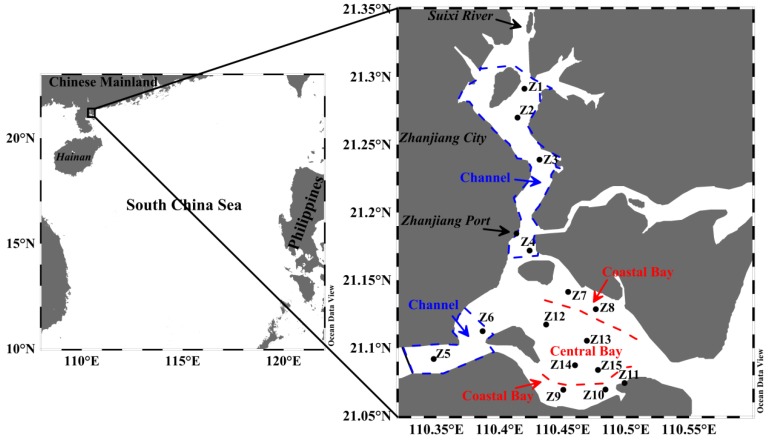
The sampling sites (Z1–Z15) in Zhanjiang Bay (ZJB), China. Zhanjiang Bay is divided into three subregions, namely the channel, coastal bay, and central bay.

Zhanjiang Bay (ZJB) is a semi-enclosed bay located in the northwestern coastal region of the South China Sea (Figure 1). It covers an area of about 490 km^2^, surrounded by an urban center (Zhanjiang City, Guangdong province) with a population of approximately 7.3 million. There are many sewage outfalls in ZJB [41]. Zhanjiang port, located in the ZJB (Figure 1), has an annual throughput of more than 70 million tons of cargo. ZJB is also among the most intensive mariculture areas in China [41]. Over recent decades, rapid economic development and urbanization have significantly impacted the ZJB environment. Eutrophication and harmful algal blooms have occurred frequently [42,43]. ZJB has a subtropical oceanic monsoon climate with higher temperatures and rainfall in summer than in spring. Primary production in this area also shows seasonal variations [44]. These anthropogenic activities and seasonal variations in the environment have the potential to alter the chemical and isotopic signatures of OM in surface sediments [45]. However, little is known about the influence of anthropogenic activities and seasonal environmental variations on the SOM of ZJB. The OM signatures of sediments can be used for interpretation of the effects of climate changes and anthropogenic activities on coastal ecosystems [34,46,47,48]. This study examined the TOC and TN contents, TOC/TN ratios, and δ^13^C and δ^15^N values in the surface sediments of ZJB during spring and summer. Spatial and seasonal variations in OM signatures were determined and used to evaluate the effects of anthropogenic activities and environmental changes on the SOM. The results of this study will help to address the gaps in the understanding of the effects of anthropogenic activities and seasonal environmental variations (especially the seasonal variation of rainfall) on the SOM of subtropical bays.

## 2. Materials and Methods 

### 2.1. Study Region

ZJB is located in the southern part of the Chinese mainland and is linked to the South China Sea by a narrow inlet that is 1.9 km in length (Figure 1). The water depths of ZJB generally range from 10 to 20 m. There are some small rivers flowing into ZJB, and the Suixi River is the largest of these (Figure 1). ZJB can be divided into three subregions, namely the channel, coastal ZJB (referred to hereafter as the “coastal bay”), and central ZJB (hereafter the “central bay”) (Figure 1). There are many sewage outlets in the channel [41]. Besides, the channel has intensive mariculture activities when compared with the other two subregions [41]. ZJB is located in a subtropical monsoon climate zone. Hurricanes and tropical storms enter ZJB between June and October. ZJB has a significant seasonal variation in rainfall. The average annual precipitation is 1567.3 mm, with about 56% of rainfall occurring during May to September. The average water temperature is about 29 °C in spring and 31 °C in summer [44]. The concentrations of chlorophyll a are relatively higher in summer than in spring [44].

### 2.2. Sampling and Analyses

Sampling was carried out in ZJB during two cruises in April (spring) and August (summer) in 2017. Fifteen stations, covering most of the bay (Figure 1), were selected for testing. The sampling sites Z1, Z2, Z3, Z4, Z5, and Z6 are located in the channel; Z7, Z8, Z9, Z10, and Z11 are located in the coastal bay; and Z12, Z13, Z14, and Z15 are located in the central bay. Surface sediment samples were collected using a stainless steel grab sampler. The sediment surface layer (0–2 cm) was collected at each sampling site [14,23,29]. After collection, the samples were homogenized and placed into sterile polyethylene bags, sealed, and kept on ice in a cooler during transport. In the laboratory, all samples were kept frozen at −20 °C until subsequent processing.

For grain-size analysis, a small portion of each sediment sample was pretreated with 30% H_2_O_2_ to remove OM, and with 1 M HCl to remove carbonates. The pretreated samples were then washed 3 times with deionized water. The solids were dispersed with 10 mL of 0.05 M (NaPO_3_)_6_ and then analyzed for grain size (between 0.02 and 2000 μm) using a Malvern Mastersizer 2000 laser diffractometer. The percentages of the clay (<4 μm), silt (4–64 μm), and sand (>64 μm) fractions were determined.

The frozen sediment samples were freeze-dried, ground, and homogenized with a pestle and mortar, then passed through a mesh sieve (150 μm in pore size) prior to analysis of the elemental concentrations and stable isotope ratios. For analysis of TOC and δ^13^C, the sediment samples were treated with a 1 M HCl solution to remove carbonates. The samples were then washed with deionized water, obtaining a neutral condition before being dried at 60 °C [49,50], homogenized with a pestle and mortar, and then weighed for the TOC and δ^13^C analyses. TN and δ^15^N were analyzed without pretreatment with HCl. The elemental and isotopic analyses were conducted at the Third Institute of Oceanography, Ministry of Natural Resources, China. Concentrations of the TOC and TN, as well as the δ^13^C and δ^15^N values, were determined using an elemental analyzer integrated with an isotope ratio mass spectrometer (Flash EA 1112 HT-Delta V Advantages, Thermo). Replicate analysis of one sample (*n* = 5) gave a relative standard deviation less than 0.8% for TOC and less than 0.7% for TN. The TOC/TN ratios presented here are the molar ratios of TOC to TN content. The δ^13^C and δ^15^N values are reported relative to the Vienna PeeDee Belemnite standard (V-PDB) and atmospheric nitrogen, respectively. Acetanilide was used as a working standard (δ^13^C = −29.53‰ and δ^15^N = 1.18‰). The instrument analytical precision was ± 0.2‰ for δ^13^C and ±0.25‰ for δ^15^N.

## 3. Results

### 3.1. Grain Size

**Figure 2:**
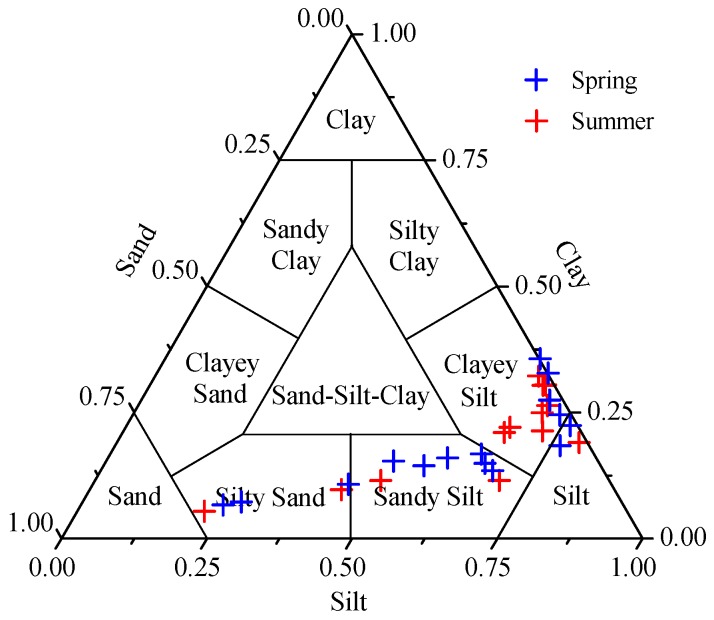
Ternary diagram showing the Shepard’s classification and textures of the surface sediments in spring and summer.

**Figure 3:**
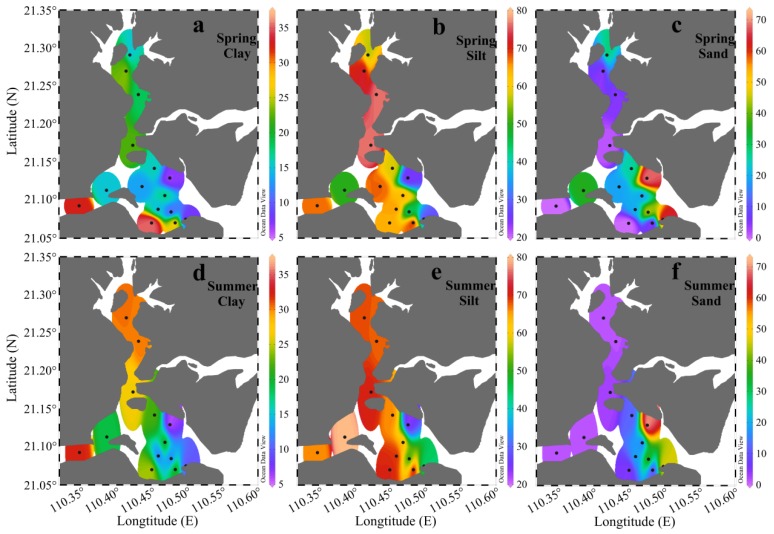
Spatial variations in the clay (**a**,**d**), silt (**b**,**e**), and sand (**c**,**f**) fractions of the surface sediments collected in Zhanjiang Bay in spring and summer (unit: %).

Grain size compositions can significantly influence the behavior of sedimentary parameters. The ternary diagram in Figure 2 categorizes the surface sediments of ZJB. It showed that the surface sediments of ZJB were predominantly composed of clayey silt and sandy silt. The surface sediments of only a few sites were composed of silt and silty sand (Figure 2). Figure 3 shows the distribution of the grain size fractions. The distribution patterns of grain size in the ZJB were similar in spring and summer. Fine-grained sediments (clay + silt) predominated in the channel, while coarser sandy sediments dominated the area near to the bay mouth (Figure 3). Considering the two sampling seasons, the percentages of clay ranged from 5.4% to 35.5%, with a mean of 19.3% ± 8.5% (mean ± SD). Silt ranged from 21.9% to 79.6%, with a mean of 60.5% ± 15.6%, and sand ranged from 0% to 72.8%, with a mean of 20.2% ± 22.3%. Across all the sampling sites, the average concentrations of the clay and silt fractions did not show obvious seasonal variations (Table 1). However, the average sand fraction in spring (22.7% ± 22.9%) was significantly higher than in summer (17.3% ± 22.2%).

Table 2 showed the mean values of the sediment parameters in different subregions of the ZJB. Based on the data of the two seasons, the clay fraction was highest in the channel (24.2% ± 6.8%), intermediate in the coastal bay (18.0% ± 10.7%), and lowest in the central bay (14.2% ± 3.7%). The silt fraction was highest in the channel (68.2% ± 9.1%), intermediate in the central bay (60.7% ± 9.9%), and lowest in the coastal bay (51.4% ± 20.2%). The sand fraction was highest in the coastal bay (30.5% ± 29.9%), intermediate in the central bay (25.0% ± 12.1%), and lowest in the channel (7.7% ± 12.6%). In general, the surface sediment in the channel had the finest grain size, with sand contributing the least to the surface sediment (Table 2). Large-scale, cage-based mariculture in the channel may be responsible for this phenomenon, because mariculture can weaken the hydrodynamic conditions and contribute to the settlement of fine particles [15,22,41,51]. Obvious seasonal variations of the grain size fractions were observed in the channel and the coastal bay (Table 2). In the channel, the average clay fraction in summer was 30.0% higher than that in spring, while the average sand fraction in summer was 83.9% lower than in spring. In the coastal bay, the average silt fraction in summer was 10.0% higher than in spring, while the average sand fraction in summer was 11.7% lower than in spring. The grain size fractions of surface sediments in the central bay did not show obvious seasonal variations (Table 2). In summary, the surface sediments in the channel and coastal bay were relatively finer in summer than in spring.

### 3.2. TOC and TN

**Figure 4:**
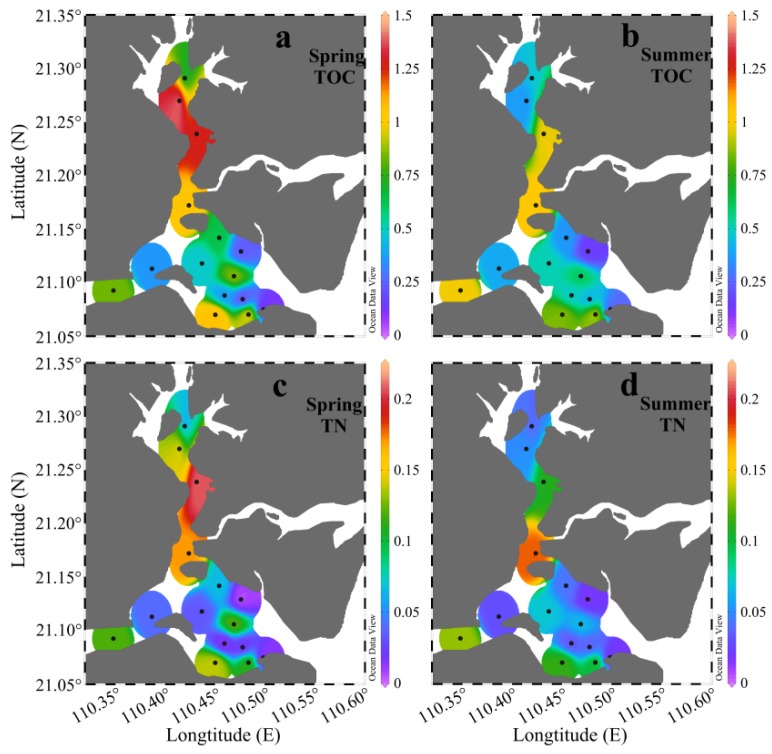
Spatial variations in the TOC (**a**,**b**) and TN (**c**,**d**) in the surface sediments of Zhanjiang Bay in spring and summer (unit: %).

The TOC in sediment represents OM that escapes remineralization [52]. It is widely used as a proxy for describing the abundance of OM [1,27]. In ZJB, the TOC in spring ranged from 0.10% to 1.46%, with an average of 0.70% ± 0.41%, while in summer it had a slightly narrow range of 0.15% to 1.04%, with an average of 0.59% ± 0.29% (Figure 4a,b). The average TOC concentration in spring was 19% higher than in summer. The distribution pattern of TOC in ZJB was similar in spring and summer, with generally higher TOC contents in the channel in both seasons (Figure 4a,b). This distribution pattern was similar to the distribution pattern of fine-grained sediment (clay and silt) (Figure 3). Significant positive relationships between fine-grained fractions and TOC concentrations were found in both spring and summer (*r* > 0.56, *P* < 0.05; Table 3). This is because the fine-grained sediments have large specific surface areas that provide good binding sites for the adsorption of OM [53,54]. In spring, the concentrations of TN ranged from 0.01% to 0.21%, with a mean of 0.08% ± 0.07%, while in summer they ranged from 0.02% to 0.18%, with a mean of 0.07% ± 0.05% (Figure 4c,d). The average concentration of TN in spring was 15% higher than in summer. As with the spatial distribution of TOC, relatively high concentrations of TN were found in the channel in both seasons (Figure 4). The TOC and TN concentrations of the surface sediment in ZJB were significantly correlated with each other in both spring and summer (*r* > 0.94, *P* < 0.001; Table 3), indicating that nitrogen was mostly present in organically bound compounds [34,55]. The differences in TOC and TN between spring and summer suggested that labile OM decomposed during the investigation period [55]. The seasonal variation pattern of TOC and TN in the surface sediment of ZJB was different from that in the coastal area of northern Shandong Peninsula (a temperate coastal area), where TOC and TN concentrations were significantly higher in summer than in spring [14]. The relatively high temperature in ZJB (a subtropical bay) is more conducive to the decomposition of OM and reduces the accumulation of OM [56]. This may be the reason for the different seasonal variation patterns of SOM in these two areas.

Based on the data from the two seasons, the TOC was highest in the channel (0.82% ± 0.37%), intermediate in the coastal bay (0.55% ± 0.38%), and lowest in the central bay (0.49% ± 0.19%) (Table 2). The TN had a similar distribution pattern with the TOC, with the highest value in the channel (0.11% ± 0.06%), an intermediate value in the coastal bay (0.06% ± 0.05%), and the lowest value in the central bay (0.05% ± 0.04%) (Table 2). Mariculture activities can strongly increase sedimentation rates and decrease hydrodynamic conditions [15,57]. This contributed to the high concentrations of the TOC and TN in the sediments of the channel and coastal bay, where there is greater maricultural activity [41]. Dredging and resuspension may be responsible for the low concentrations of TOC and TN in the central bay [27,58]. This will be discussed further in Section 4.1.1. Obvious seasonal variations in the TOC and TN were observed in the channel and coastal bay, with the average TOC and TN concentrations being higher in spring and lower in summer (Table 2). The average water temperature in ZJB is about 29 °C in spring and 31 °C in summer [44]. Higher temperature is more conducive to the decomposition of OM [56]. This may be the reason why the concentrations of TOC and TN were low in the channel and coastal bay in summer. In the central bay, OM may be relatively refractory (as discussed later). No obvious seasonal variations in TOC and TN were observed in this subregion (Table 2).

### 3.3. δ^13^C, ^δ15^N, and TOC/TN

**Figure 5:**
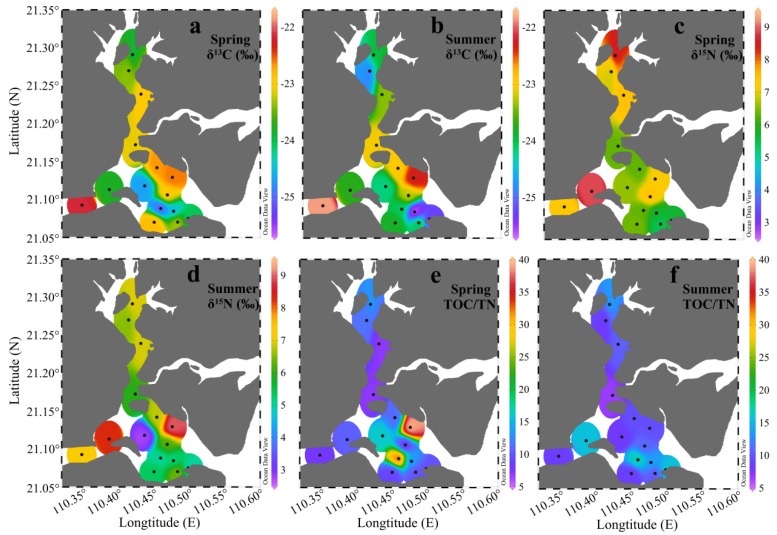
Distributions of δ^13^C (**a**,**b**), δ^15^N (**c**,**d**), and TOC/TN (**e**,**f**) in the surface sediments of Zhanjiang Bay in spring and summer.

Figure 5a–d showed the spatial distributions of δ^13^C and δ^15^N in the surface sediments of ZJB in spring and summer. In spring, the values of δ^13^C ranged from −25.0‰ to −22.2‰, with a mean of −23.4‰ ± 0.8‰. In summer, they ranged from −25.7‰ to −21.8‰, with a mean of −23.7 ± 1.0‰. Overall, the spatial distributions and the values of δ^13^C in the two seasons were rather similar (Figure 5a,b). In spring, the values of δ^15^N ranged from 5.4‰ to 8.9‰, with a mean of 6.8‰ ± 1.0‰. In summer, they ranged from 2.8‰ to 9.0‰, with a mean of 6.2‰ ± 1.5‰. The average value of δ^15^N in spring was about 10% higher than in summer, showing an obvious seasonal variation. In both seasons, relatively high δ^15^N values were found in the channel (Figure 5c,d). Figure 5e,f showed the spatial distributions of the TOC/TN molar ratios in spring and summer, respectively. The TOC/TN ratios ranged from 7.1 to 39.4 in spring, with a mean of 13.8 ± 9.4, and from 6.7 to 17.2 in summer, with a mean of 11.2 ± 3.3. The average molar ratio of TOC/TN in spring was 23% higher than in summer.

As shown in Table 2, the average δ^13^C value in spring and summer was highest in the channel (−23.3‰ ± 0.8‰), intermediate in the coastal bay (−23.4‰ ± 0.9‰), and lowest in the central bay (−24.3‰ ± 0.9‰). Here, δ^15^N showed a similar distribution pattern, with the highest value in the channel (7.3‰ ± 0.9‰), an intermediate value in the coastal bay (6.4‰ ± 1.2‰), and the lowest value in the central bay (5.7‰ ± 1.4‰) (Table 2). The value of TOC/TN showed a converse distribution pattern to δ^13^C and δ^15^N, with the highest average TOC/TN value being found in the central bay (15.2 ± 7.8), an intermediate average of TOC/TN in the coastal bay (13.3 ± 9.4), and the lowest average of TOC/TN in the channel (10.1 ± 3.0) (Table 2). Additionally, δ^13^C, δ^15^N, and TOC/TN also showed some seasonal variations in the different subregions. The environmental indications of TOC/TN, δ^13^C, and δ^15^N, and the reasons for their spatial and seasonal variations are discussed below.

## 4. Discussion

### 4.1. Environmental Indications of TOC/TN, δ^13^C, and δ^15^N

#### 4.1.1. Environmental Indications of TOC/TN

TOC/TN ratios have often been used for tracing OM sources in sediment samples [14,59,60]. In general, terrestrial OM and marine OM have TOC/TN ratios of >15 and ~5–8, respectively [61]. The TOC/TN ratios of the surface sediment in ZJB ranged from 6.7 to 39.4, with the lowest value at station Z4 in summer, and the highest value at station Z8 in spring (Figure 5e,f). This indicated that the SOM in ZJB was influenced by both terrestrial and marine OM. The TOC/TN ratios of the surface sediments in ZJB obtained in this study were comparable with those of the Pearl River Estuary [18,62] and the Beibu Gulf [8], but generally higher than those of the East China Sea shelf [63], Sishili Bay [34], Bohai Bay [64], and the Changjiang Estuary and adjacent sea [65] (Table 4). Table 2 showed the average TOC/TN ratios in different subregions in different seasons. Based on the data of spring and summer, the average TOC/TN ratio was highest in the central bay (15.2 ± 7.8), intermediate in the coastal bay (13.3 ± 9.4), and lowest in the channel (10.1 ± 3.0). The lower average TOC/TN ratio in the channel, compared with the other two subregions, may indicate that the SOM in the channel was more influenced by marine phytoplankton. Interestingly, the δ^13^C values also indicated an increase in marine-derived OM in the channel sediment, which is discussed later.

The TOC/TN ratios of OM can be altered by postdepositional processes [61]. Early diagenesis can increase TOC/TN ratios in OM [61]. Fresh phytoplankton usually presents TOC/TN ratios of approximately 7.0–7.5, while detritus usually presents TOC/TN ratios of 20–30 [71]. In the surface sediments of ZJB, the TOC/TN ratios had no significant relationships with δ^13^C (Table 3). This could be attributed to the influence of decomposition [72]. Dredging can partially expose surface sediments to solar heating, which can severely degrade OM [27,73]. In addition, dredging can resuspend the finer sediment fraction, which is rich in TOC and can consequently affect TOC preservation [27]. The central bay, where the navigation channel is located, is affected by dredging. This may be one of the reasons why the TOC/TN ratios in the central bay were relatively high. Consistent with this, the lower average TOC and TN concentrations in the central bay (Table 2) implied a severe degradation of OM. High average TOC/TN ratios were observed in spring in the coastal and central bays (Table 2). This seasonal variation may be also related to the diagenesis of OM. According to the report of Lesen [23], SOM with high TOC/TN ratios may be more refractory. Low TOC/TN ratios may indicate high primary production [74]. This indicated that the SOM in the coastal and central bays in spring may be more refractory. Relatively fresher OM may accumulate in the sediment of coastal and central bays during summer, when the primary production is high [44]. In the channel, the average TOC/TN ratio was higher in summer than in spring, which was contrary to the findings in the coastal and central bays. This indicated that the seasonal variation of TOC/TN in the channel was more influenced by the terrestrial inputs than primary production. It is reasonable that high rainfall in summer in the ZJB area could bring about more terrestrial OM, which has higher TOC/TN values than marine OM [61,75].

Anthropogenic activities (such as the widespread use of organic chemicals) may also alter the TOC/TN ratios in SOM [9]. For the surface sediment in ZJB, the TOC/TN values had significant negative correlations with TN concentrations in both spring and summer (*r* < −0.61, *P* < 0.05; Table 3). This may reflect the disturbance from anthropogenic nitrogen inputs. High TN concentrations contribute to low TOC/TN ratios. In other words, the anthropogenic inputs of nitrogen could increase marine primary production, and thus increase marine OM inputs to the surface sediment in ZJB. The sediment grain sizes may also influence TOC/TN ratios. A study by Lesen [23] indicated that a sandy sediment quality contributed to low sediment nitrogen levels, which resulted in high TOC/TN ratios. Prahl et al. [70] also found that OM associated with coarse particles displayed high TOC/TN ratios. This phenomenon has also been found in the present study. The spring samples of stations Z8 and Z14 had high sand fractions and low TN concentrations, resulting in high TOC/TN ratios (Figure 1, Figure 3, Figure 4 and Figure 5e,f).

#### 4.1.2. Environmental Indications of δ^13^C

Terrestrial plants with a C_3_ pathway have δ^13^C values ranging from −30‰ to −24‰ [10], while terrestrial plants with a C_4_ pathway have δ^13^C values ranging from −16‰ to −10‰ [76]. Marine OM has δ^13^C values ranging from −22‰ to −18‰ [77]. In general, OM derived from terrestrial C_3_ plants has depleted δ^13^C values compared to marine OM. Diagenetic effects on the δ^13^C signature of OM have been shown to be small [78,79]. Therefore, δ^13^C is a perfect tool for distinguishing different OM sources [14,66], and it has been widely used to distinguish between terrestrial and marine sources of OM [14,19,66].

C_4_ plants are uncommon in the area surrounding ZJB, where the dominant cultivated plant is rice (a C_3_ plant) and the natural ecosystem is a subtropical forest. Therefore, the contribution of C_4_ plants to the SOM in ZJB can be neglected [66,80]. Taking the results of the spring and summer samples as a whole, the δ^13^C values in the surface sediments of ZJB ranged from −25.7‰ to −21.8‰ (Figure 5a,b and Table 4), which was generally within the δ^13^C range covering marine-derived and terrestrial-derived OM [76,77]. This indicated a mix of terrestrial and marine sources for the SOM in ZJB. Compared with the other areas listed in Table 4, the δ^13^C values in ZJB obtained in this study were comparable to those of the Pearl River Estuary [18], the Changjiang Estuary [68,69,81], the coastal Bohai Bay area [9], and the Washington continental shelf [70], but lower than those of Ailian Bay [15], Zheling Bay [40], the East China Sea shelf [63], and coastal areas of the East China Sea [67] (Table 4).

Table 2 shows the average δ^13^C values of the different subregions. The average δ^13^C value was highest in the channel (−23.3‰ ± 0.8‰), intermediate in the coastal bay (−23.4‰ ± 0.9‰), and lowest in the central bay (−24.3‰ ± 0.9‰), suggesting that the channel and the coastal bay were more influenced by marine phytoplankton than the central bay. According to previous studies, δ^13^C values of SOM in coastal sediments generally increase in a seaward direction [8,64,77]. The reason for this is that the relative proportion of terrigenous OM decreases in a seaward direction. However, this distribution pattern is not evident in this study. We believe that the distribution pattern of δ^13^C in this study was influenced by human activities. Relatively high δ^13^C values have also been seen in the coastal area of northern Shandong Peninsula [14]. The channel and the coastal bay had high concentrations of nutrients as a result of human activities, such as agriculture, mariculture, and industry in or around ZJB [41,58,82]. Marine phytoplankton may utilize these nutrients, and thus high primary production could occur [83], which could result in a high contribution of marine phytoplankton to the sediments in these areas [8]. The increased TOC and TN concentrations and decreased TOC/TN ratios in the channel and the coastal bay (Table 2) also indicated increased primary production in these two subregions [27,37]. In the central bay, the contribution of marine OM to the sediment may be limited by relatively low nutrient concentrations or by high turbidity in the water column caused by numerous large ships frequently passing by [84,85,86,87].

Compared with the δ^13^C values in the channel and coastal bay in spring, the δ^13^C values in these two subregions in summer were relatively negative (Table 2). Such seasonal variations may be explained by the greater contribution of ^13^C-depleted OM in summer. Heavy summer rainfall [75] can bring large amounts of terrestrial OM into the channel and the coastal bay, reducing the δ^13^C values in the surface sediments there. The average δ^13^C value in the central bay was similar in spring and summer (Table 2). The reason for this may be that terrestrial input was limited to the channel and the coastal bay; the central bay was less influenced by terrestrial input because of its greater distance from the coast (Figure 1).

The δ^13^C value of SOM in the central bay obtained in this study (−24.3‰ ± 0.9‰ in spring and −24.3‰ ± 1.0‰ in summer) generally showed greater depleted values and less seasonal variability than particulate OM obtained in the same area by Cai [44] (−23.9‰ ± 1.7‰ in spring and −20.5‰ ± 2.0‰ in summer). This phenomenon has also been found in a western Mediterranean coastal lagoon [88].

#### 4.1.3. Environmental Indications of δ^15^N

The δ^15^N value can also be used to determine the sources of SOM [14,36,89]. The δ^15^N values of marine OM usually range from 4‰ to 10‰ (mean 6‰), while the δ^15^N values of terrestrial OM usually range from −10‰ to 10‰ (mean 2‰) [90]. Although the marine component has relatively higher δ^15^N values than terrestrial OM [61,91], the δ^15^N values of these two sources overlap to some extent. Also, the δ^15^N values of OM could potentially be modified by selective diagenesis during sinking and after sedimentation [92]. Therefore, δ^15^N does not discriminate as effectively as δ^13^C between terrestrial and marine OM sources. However, compared to δ^13^C, δ^15^N can be effectively used to distinguish between sewage inputs and other sources because of their different δ^15^N values [9,10,93]. Nitrogen delivered from agricultural runoff and human sewage has relatively high δ^15^N values (10‰–22‰) [69,94]. Marine plants in proximity to sewage outlets can absorb and assimilate sewage-derived nitrogen, resulting in high δ^15^N values (~10‰) in their tissue [16]. The settlement of these marine plants may then result in high δ^15^N values in the sediment. Aquaculture could also lead to elevated δ^15^N values in the sediment (δ^15^N > 7‰) [89]. The use of δ^13^C in identifying sewage inputs is limited, because the δ^13^C values of sewage effluents (which vary from −26‰ to −22‰) overlap with the values of terrestrial and marine sources [10].

In this study, the δ^15^N values for the complete set of samples in ZJB ranged from 2.8‰ to 9.0‰ (Table 4), which was within the ranges of δ^15^N values of marine phytoplankton and terrestrial OM. This indicated the existence of mixed sources in the surface sediment of ZJB. The values of δ^15^N in ZJB obtained in this study were higher than those of the East China Sea shelf [63] and the Pearl River Estuary [66], but were comparable to those of Ailian Bay [15] and the Beibu Gulf [8] (Table 4). Table 2 summarizes the average δ^15^N values in the different subregions. The average δ^15^N value was highest in the channel, intermediate in the coastal bay, and lowest in the central bay. According to Ke et al. [41], there are many sewage outlets and a large amount of maricultural activity in the channel. The δ^15^N values in the channel (which were generally higher than 7‰; Figure 5c,d; Table 2) were generally higher than those of the terrestrial OM (mean 2‰) and the marine OM (mean 6‰), but close to the values of OM derived from aquaculture and marine plants in proximity to sewage outlets (>7‰) [16,89,90]. This indicated that the channel was more influenced by anthropogenic activities. This result was consistent with the relatively high TOC and TN concentrations in the channel (Table 2), which were indicative of anthropogenic inputs [95]. 

Clear seasonal variations in δ^15^N values were observed in the central bay. The average δ^15^N value in spring (6.5‰ ± 0.7‰) was significantly higher than in summer (4.8‰ ± 1.4‰) (Table 2). The decreased δ^15^N values in summer in the central bay may be related to the changes in the nitrogen used by phytoplankton [88]. In winter and early spring, phytoplankton utilizes “new” nitrogen in the form of nitrate, which is more enriched in ^15^N [96,97]. In summer, much of the available nitrate has been used, and marine phytoplankton utilizes more recycled ammonia, which is generally depleted in ^15^N [97]. Therefore, the settlement of dead phytoplankton with different δ^15^N values in different seasons may be responsible for the seasonal variations in sedimentary δ^15^N values in the central bay. In the channel, the average δ^15^N value in summer was slightly lower than in spring. High rainfall in summer, which can bring substantial terrestrial OM with relatively low δ^15^N values, may be responsible for this phenomenon [75,91].

### 4.2. Quantification of Terrestrial and Marine Organic Matter Sources

Diagenesis can affect the chemical and isotopic compositions of OM, especially in the case of δ^15^N and TOC/TN [55,66]. However, such fractionation was much smaller than the source differences for δ^13^C [98]. In ZJB, the TOC/TN did not show a significant correlation with δ^13^C in either spring or summer (Table 3). Additionally, δ^15^N showed significant positive correlations with δ^13^C in summer, while this was not the case in spring (Table 3). Therefore, only δ^13^C was used for the quantification analysis of OM. The relative proportions of terrestrial OM (f_ter_) and marine OM (f_mar_) in the sediments of ZJB can be assessed using the δ^13^C-based two end-member mixing model [14,40]. The equations which the two end-member mixing model is based on are as follows:

(1) δ^13^C_sample_ = δ^13^C_ter_ × f_ter_ + δ^13^C_mar_ × f_mar_

(2) f_ter_ + f_mar_ = 1

where δ^13^C_ter_ and δ^13^C_mar_ are the δ^13^C values of the terrestrial and marine OM end-members, respectively. Due to the lack of δ^13^C values for the terrestrial and marine end-members for the SOM in ZJB, we chose the δ^13^C value of C_3_ plants (average: −27‰) as the terrestrial end-member [61,76]. For the marine end-member, the δ^13^C value of phytoplankton (−20.8‰), collected from the northern South China Sea, was chosen [18].

**Figure 6:**
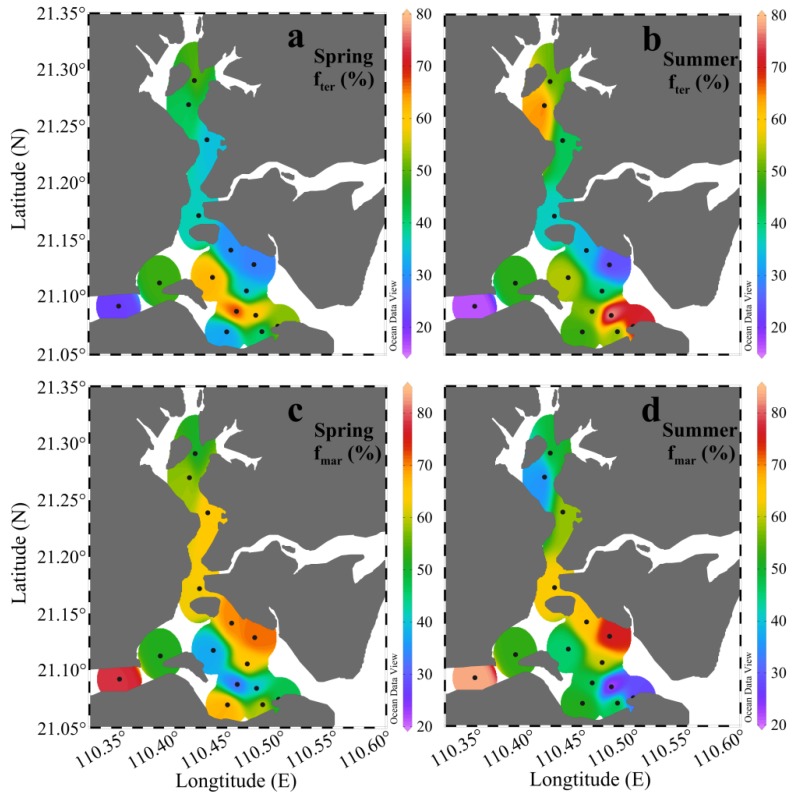
The percentage distribution of terrestrial (f_ter_) and marine (f_mar_) organic matter in the surface sediments of Zhanjiang Bay in spring and summer. (**a**): the percentage distribution of terrestrial organic matter in spring; (**b**): the percentage distribution of terrestrial organic matter in summer; (**c**): the percentage distribution of marine organic matter in spring; (**d**): the percentage distribution of marine organic matter in summer.

The results from the δ^13^C-based two end-member mixing model are displayed in Figure 6. Taken as a whole, the surface sediment in ZJB was dominated by marine-derived OM (average f_mar_: 54.9% ± 15.2%). In spring, the calculated f_ter_ ranged from 22.1% to 68.2%, with a mean of 42.8% ± 13.7%. In summer, it varied from 16.4% to 78.4%, with a mean of 47.5% ± 16.8%. The relatively higher average f_ter_ value in the summer could probably be related to the high rainfall in this season, which could bring large amounts of terrestrial OM into ZJB [75]. The average f_ter_ value of the different subregions in different seasons confirmed this conclusion, which is discussed below.

**Figure 7:**
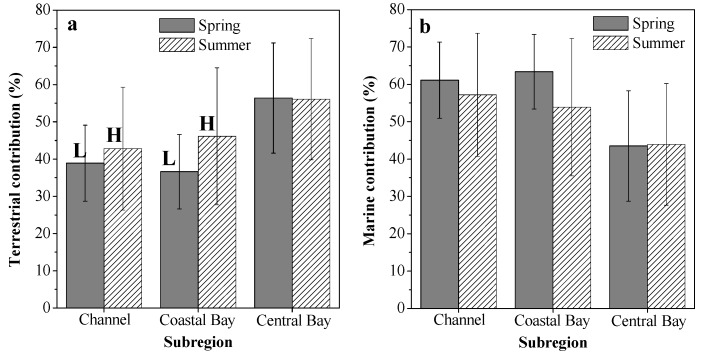
Source contributions of terrestrial (**a**) and marine (**b**) organic matter for the surface sediments in different subregions of Zhanjiang Bay. Different capital letters in Figure 7a indicate obvious seasonal variations with average seasonal differences larger than 10%.

In different subregions, the calculated f_ter_ and f_mar_ values also showed spatial and seasonal variations, as summarized in Figure 7. Relatively high proportions of marine OM were observed in the channel and coastal bay in both spring and summer (Figure 7b). This may indicate that maricultural activities and sewage input had significant influences on SOM sources in the channel and coastal bay. Indeed, large amounts of nutrients from maricultural activity and sewage input have been brought into the channel and coastal bay [41]. This can stimulate primary production in these two subregions, resulting in relatively high proportions of marine OM in their sediments. This finding has important implications for OM distributions in coastal regions under severe influences from human activities. Many other studies also have found that mariculture activities and terrestrial input can stimulate algal blooms and cause increases in marine OM in coastal sediments [15,34,40]. Clear seasonal variations in the average f_ter_ value were observed in the channel and coastal bay (Figure 7a). The average f_ter_ values in summer increased by 10.2% in the channel and by 26.0% in the coastal bay. With greater summer rainfall [75], large amounts of terrestrial OM are transported from the surrounding area of ZJB by rain floods and then deposited in the channel and coastal bay, resulting in relatively high proportions of terrestrial OM in summer in these two subregions (Figure 7a). High summer rainfall is a common characteristic for the subtropical bays in the coastal northern South China Sea. Therefore, we think that the high input of terrestrial OM in summer may also exist for other subtropical bays in the coastal northern area of the South China Sea. More investigations of seasonal variations of SOM in subtropical bays should be conducted to confirm this conclusion.

## 5. Conclusions

This study investigated the spatial and seasonal variations of OM in the surface sediments of ZJB. TOC, TN, TOC/TN, δ^13^C, and δ^15^N were analyzed to identify the effects of anthropogenic activities and environmental changes on SOM signatures in this area. Based on combined δ^13^C, δ^15^N, and TOC/TN information, we concluded that the OM in the surface sediments of ZJB was a mixture of terrestrial and marine sources. A δ^13^C-based two end-member mixing model indicated that the surface sediments in ZJB were dominated by marine-derived OM (average proportion of 54.9% ± 15.2%).

Anthropogenic activities had a significant influence on the distribution patterns of TOC, TN, TOC/TN, δ^13^C, and δ^15^N in the surface sediments of ZJB. Compared to the central bay, relatively higher δ^13^C and δ^15^N values, relatively lower TOC/TN values, and relatively higher TOC and TN concentrations were found in the sediments in the channel and coastal bay. This indicated elevated primary productivity and relatively high marine OM in these areas. Nutrients from nearby sewage outlets, rapid urbanization, mariculture activities, and heavily fertilized farming in the surrounding area were found to be responsible for these phenomena. Dredging caused subaerial exposure of the surface sediment and affected OM preservation in the central bay. Lower δ^13^C and δ^15^N values and higher TOC/TN ratios, together with the lower TOC and TN concentrations in the central bay sediments, indicated the low-efficiency storage of marine OM in this area.

Seasonal variations in SOM parameters were observed in ZJB, with the TOC and TN concentrations and δ^13^C values being higher in spring in the channel and coastal bay, and the TOC/TN ratios being higher in spring in the coastal and central bays. Additionally, the δ^15^N values were higher in spring in the channel and central bay. Higher temperatures were responsible for the low concentrations of TOC and TN in the channel and coastal bay in summer. Heavy summer rainfall increased the input of terrestrial OM in the channel and coastal bay, resulting in a decrease in δ^13^C values in this season. Relatively low TOC/TN ratios in summer in the coastal and central bays may be related to the accumulation of fresh OM in this season when primary production was high. High rainfall in summer was also responsible for low δ^15^N values in this season in the channel, while the low δ^15^N values in summer in the central bay may be related to the changing sources of nitrogen used by phytoplankton.

The results of this study indicate that large amounts of terrestrial OM can be brought to coastal sediment areas during summer when rainfall is high. The decomposition of SOM can consume a large amount of oxygen in water. This indicates that high rainfall may aggravate the risk of hypoxia for the bottom water in coastal areas through increasing terrestrial input. Therefore, measures should be taken to reduce the input of terrestrial OM during high rainfall. More investigations should be conducted to study the influence of terrestrial OM during high rainfall periods for the protection of coastal environments.

## Figures and Tables

**Table 1:** Mean values (mean ± SD) of the grain size fractions for the full sampling sites in the Zhanjiang Bay in spring and summer.

Season	Clay (%)	Silt (%)	Sand (%)
Spring	18.4 ± 8.6	58.9 ± 16.2	22.7 ± 22.9
Summer	20.4 ± 8.7	62.3 ± 15.4	17.3 ± 22.2

**Table 2:** Mean values (mean ± SD) of the carbon and nitrogen parameters and grain size compositions for the surface sediments in different subregions of the Zhanjiang Bay. TOC, total organic carbon; TN, total nitrogen; δ^13^C, stable carbon isotope composition; δ^15^N, stable nitrogen isotope composition.

Season	Subregion	TOC (%)	TN (%)	TOC/TN	δ^13^C (‰)	δ^15^N (‰)	Clay (%)	Silt (%)	Sand (%)
Spring	Channel	**0.94 ± 0.41**	**0.12 ± 0.07**	**9.6 ± 2.5**	−23.2 ± 0.6	7.6 ± 1.0	**21.3 ± 6.8**	66.4 ± 11.5	**12.4 ± 16.0**
	Coastal Bay	**0.60 ± 0.43**	**0.07 ± 0.06**	**15.8 ± 13.2**	−23.1 ± 0.6	6.2 ± 0.7	18.5 ± 12.7	**49.0 ± 21.5**	**32.4 ± 33.2**
	Central Bay	0.48 ± 0.28	0.05 ± 0.05	**17.7 ± 10.5**	−24.3 ± 0.9	**6.5 ± 0.7**	13.9 ± 2.5	60.2 ± 10.9	25.9 ± 12.9
Summer	Channel	**0.71 ± 0.33**	**0.09 ± 0.06**	**10.6 ± 3.6**	−23.5 ± 1.0	7.0 ± 0.8	27.7 ± 5.3	70.3 ± 5.4	**2.0 ± 0.7**
	Coastal Bay	**0.50 ± 0.35**	**0.06 ± 0.05**	**10.8 ± 2.9**	−23.7 ± 1.1	6.5 ± 1.6	17.5 ± 9.8	**53.9 ± 21.0**	**28.6 ± 30.1**
	Central Bay	0.51 ± 0.07	0.05 ± 0.02	**12.6 ± 3.8**	−24.3 ± 1.0	**4.8 ± 1.4**	14.7 ± 5.5	61.5 ± 10.8	23.8 ± 13.7
Spring and Summer	Channel	0.82 ± 0.37	0.11 ± 0.06	10.1 ± 3.0	−23.3 ± 0.8	7.3 ± 0.9	24.2 ± 6.8	68.2 ± 9.1	7.7 ± 12.6
	Coastal Bay	0.55 ± 0.38	0.06 ± 0.05	13.3 ± 9.4	−23.4 ± 0.9	6.4 ± 1.2	18.0 ± 10.7	51.4 ± 20.2	30.5 ± 29.9
	Central Bay	0.49 ± 0.19	0.05 ± 0.04	15.2 ± 7.8	−24.3 ± 0.9	5.7 ± 1.4	14.2 ± 3.7	60.7 ± 9.9	25.0 ± 12.1

Bold values indicate obvious seasonal variations (with seasonal differences larger than 10%).

**Table 3:** Relationship between sediment carbon and nitrogen parameters and sediment grain-size fractions in spring and summer.

Season	Variables	Clay	Silt	Sand	TOC	TN	TOC/TN	δ^13^C	δ^15^N
Spring	TOC	**0.731 ^b^**	**0.811 ^c^**	**−0.848 ^c^**	1				
	TN	**0.689 ^b^**	**0.761 ^b^**	**−0.797 ^c^**	**0.939 ^c^**	1			
	TOC/TN	−0.482	−0.461	0.507	−0.505	**−0.611 ^a^**	1		
	δ^13^C	0.497	0.131	−0.279	0.497	**0.572 ^a^**	−0.225	1	
	δ^15^N	−0.064	0.006	0.019	0.053	0.064	−0.026	0.161	1
Summer	TOC	**0.704 ^b^**	**0.564 ^a^**	**−0.665 ^b^**	1				
	TN	**0.720 ^b^**	0.467	**−0.604 ^a^**	**0.935^c^**	1			
	TOC/TN	**−0.641 ^a^**	−0.086	0.309	−0.456	**−0.670 ^b^**	1		
	δ^13^C	0.294	0.015	−0.125	0.352	0.339	−0.426	1	
	δ^15^N	0.042	−0.188	0.114	−0.082	−0.098	0.002	**0.544 ^a^**	1

Bold values indicate significant correlations at *P* < 0.05; ^a^ 0.01 < *P* < 0.05; ^b^ 0.001 < *P* < 0.01; ^c^ *P* < 0.001.

**Table 4:** Comparison of δ^13^C, δ^15^N, and TOC/TN ratios in the sediments of Zhanjiang Bay with related data.

Location	Sampling Time		δ^13^C (‰)	δ^15^N (‰)	TOC/TN	Reference
Zhanjiang Bay	Apr., 2017	Range	−25.0 to −22.2	5.4–8.9	7.1–39.4	This study
		Mean	-23.4 ± 0.8	6.8 ± 1.0	13.8 ± 9.4	
	Aug., 2017	Range	−25.7 to −21.8	2.8–9.0	6.7–17.2	This study
		Mean	−23.7 ± 1.0	6.2 ± 1.5	11.2 ± 3.3	
	Apr. and Aug., 2017	Range	−25.7 to −21.8	2.8–9.0	6.7–39.4	This study
		Mean	−23.6 ± 0.9	6.6 ±1.3	12.5 ± 7.1	
Beibu Gulf	Jul., 2010	Range	−27.8 to −20.0	0.8–9.0	2.2–33.3	[8]
Pearl River Estuary	Spring, 2003	Range	−25.19 to −23.33	3.73–6.57	8.1–17.2	[66]
	Mar., 2005	Range	na ^a^	na	8.50–15.32	[62]
		Mean	−24.3	na	na	
	2001–2005	Range	−26.1 to −21.3	na	6.5-22.4	[18]
Zheling Bay	Sep., 2013	Range	−22.07 to −21.13	5.28–7.14	6.43–8.20	[40]
East China Sea Shelf	May to Jun., 2014	Range	−22.08 to −19.99	3.67–6.28	4.69–9.12	[63]
		Mean	−21.35	4.60	6.74	
	Oct. to Nov., 2014	Range	−21.97 to −20.10	4.60–6.13	4.78–8.89	[63]
		Mean	−21.00	5.32	6.73	
Coastal Areas of the East China Sea	Jun., 2010	Range	−21.8 to −20.7	na	na	[67]
Changjiang Estuary	na	Range	−29.8 to −23.7	1.0–7.8	na	[68]
Changjiang Estuary and Adjacent Sea	2006–2007	Range	−23.8 to 20.7	na	na	[69]
	Jul. to Aug., 2001	Range	−23.6 to −20.4	na	6.4–7.9	[65]
		Mean	−22.5	na	7.1	
Ailian Bay	Aug., 2016	Range	−19.75 to −18.50	6.59–7.32	6.29–8.39	[15]
Sishili Bay	Nov., 2008	Range	−22.7 to −21.6	5.4–6.5	7.9–10.1	[34]
Bohai Bay	2006	Range	−23.9 to −21.7	na	3.3–7.7	[64]
Coastal Bohai Bay	May, 2008	Range	−25.69 to −18.23	na	10.8–42.6	[9]
		Mean	−22.86 ± 1.43	4.05 ± 0.53	21.3 ± 6.1	
Washington Continental Shelf		Range	−25.5 to −22.6	na	12.1–17.5	[70]
		Mean	−23.9 ± 0.9	na	14.5 ± 1.6	

Note: ^a^ na, not available.
